# Synthesis and characterization of Sant-75 derivatives as Hedgehog-pathway inhibitors

**DOI:** 10.3762/bjoc.8.94

**Published:** 2012-06-06

**Authors:** Chao Che, Song Li, Bo Yang, Shengchang Xin, Zhixiong Yu, Taofeng Shao, Chuanye Tao, Shuo Lin, Zhen Yang

**Affiliations:** 1 Laboratory of Chemical Genomics, School of Chemical Biology and Biotechnology, Peking University Shenzhen Graduate School, Shenzhen 518055, China; 2 Shenzhen Shengjie Biotech Co., Ltd., Shenzhen 518055, China; 3 Department of Molecular, Cell and Developmental Biology, University of California, Los Angeles, USA; 4 Key Laboratory of Bioorganic Chemistry and Molecular Engineering of Ministry of Education and Beijing National Laboratory for Molecular Science (BNLMS), College of Chemistry, Peking University, Beijing 100871, China

**Keywords:** chemical diversity, diversity-oriented, hedgehog pathway, inhibitor, Sant-75, synthesis

## Abstract

Sant-75 is a newly identified potent inhibitor of the hedgehog pathway. We designed a diversity-oriented synthesis program, and synthesized a series of Sant-75 analogues, which lays the foundation for further investigation of the structure–activity relationship of this important class of hedgehog-pathway inhibitors.

## Introduction

**Figure 1:**
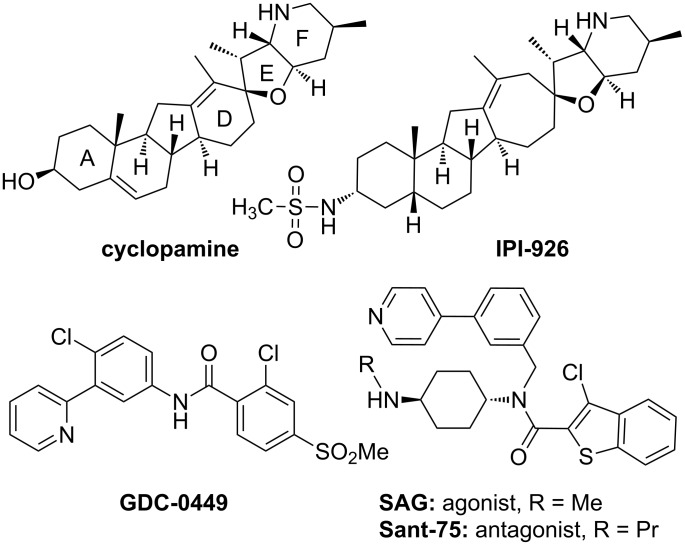
Structures of Smo antagonists and agonists.

The Hedgehog (Hh) signaling pathway plays an essential role in embryonic development and adult tissue homeostasis in metazoans. The Hh ligands activate pathway signaling by binding to a 12-transmembrane protein receptor Patched (Ptch). In the unbound state, the Ptch receptor inhibits the activity of the downstream seven-pass transmembrane receptor Smoothened (Smo). Binding of Hh ligands to Ptch leads to the alleviation of this inhibition and eventually triggers activation of the glioma (Gli) family of transcription factors and their translocation to the nucleus. This activation results in the expression of specific genes that promote cell proliferation and differentiation [1–2]. The role of the Hh signaling pathway in human cancers was first confirmed through a study of Gorlin syndrome predisposing to basal cell carcinoma, arising from autosomal dominant mutations in Ptch [3]. Indeed, aberrant Hh signaling has been reported in a variety of other malignancy diseases, such as small-cell lung cancer, pancreatic cancer, prostate cancer, breast cancer and multiple myeloma [4–9]. Taken together, the development of Hh pathway antagonists has thus represented an attractive strategy for anticancer therapy [10–11]. Because mutated Ptch or Smo proteins are mostly responsible for the abnormal activation of Hh related to human diseases, intense efforts have been invested to identify therapeutic inhibitors acting on the Smo protein. Cyclopamine (Figure 1), a natural alkaloid isolated from *Veratrum californicum* [12–13], was disclosed as the first small molecule inhibitor of the Hh pathway through direct interaction with Smo [14–15]. Cyclopamine can effectively induce a decrease in proliferation and an increase of apoptosis in several murine models [16–17]. However, the clinical development of cyclopamine as a therapeutic in cancer is hampered by its poor aqueous solubility (ca. 5 µg/mL) and acid lability. Subsequently, Infinity Pharmaceuticals developed cyclopamine-based Smo inhibitors IPI-926 through structural modification on the A and D rings. IPI-926 exhibited improved pharmaceutical properties as well as a favorable pharmacokinetic profile, and showed complete tumor regression in a Hh-dependent medulloblastoma allograft model [18–19]. IPI-926 is currently being evaluated in the phase II trial of a safety and efficacy study of patients with metastatic or locally advanced (unresectable) chondrosarcoma and myelofibrosis [19–20]. Vismodegib (GDC-0449) [21–24], developed by Genentech and Curis, is another Smo antagonist which is progressing into the phase II clinical trial for the treatment of various cancers, including advanced basal cell carcinoma, and metastatic colorectal and ovarian cancers [25–27]. Recently, vismodegib was approved by the U.S. FDA to treat adult patients with basal cell carcinoma. In addition, a number of man-made inhibitors with a Smo binding affinity have been identified and reported [2,28–34], and some of them have entered phase I development.

SAG is a synthetic Hh pathway agonist that directly targets Smo in a manner that antagonizes cyclopamine action, and thus it may serve as an interesting scaffold for drug development [35–36]. Recently, we have identified a Smo antagonist Sant-75 through zebrafish-based screening of a SAG-derived chemical library [37]. Interestingly, this antagonist differs from agonist SAG only in the chain length of the secondary alkylamine due to the different conformational changes induced. This promising result prompted us to further investigate the structure–activity relationships (SAR) of Sant-75. Herein we describe our efforts in the development of synthetic methods for the construction of a library of Sant-75.

## Results and Discussion

### Chemistry

**Scheme 1:**
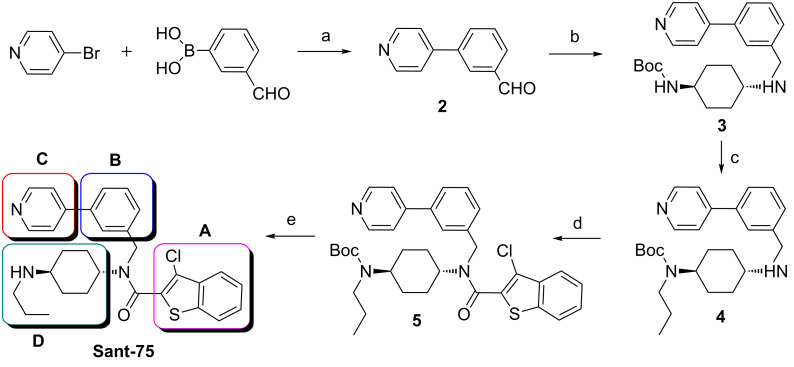
General synthetic route for Sant-75. Reagents and conditions: (a) Pd(PPh_3_)_4_, PhMe, Na_2_CO_3_, H_2_O, 85 °C; (b) *N*-Boc-cyclohexane-1,4-diamine, THF, NaBH(OAc)_3_; (c) DMF, NaH, PrI, 0 °C to rt.; (d) 3-chlorobenzo[*b*]thiophene-2-carbonyl chloride, CH_2_Cl_2_, Et_3_N; (e) CH_2_Cl_2_, TFA.

The scaffold of Sant-75 is divided into four distinct parts, namely 3-chlorobenzothiophene (motif A), a phenyl ring (motif B), 4-pyridine (motif C) and *N*-propyl-cyclohexane-1,4-diamine (motif D) (Scheme 1). In our earlier studies, the nature of the substituents on these regions was shown to have a profound effect on the activity. Examples of substituents that impart favorable activity include the alkyl group in region D.

By modification of our first generation of synthetic methodology [38], the new general synthesis of the derivatives of Sant-75 is illustrated in Scheme 1. Accordingly, Suzuki coupling of 4-bromopyridine and 3-formylphenylboronic acid afforded biaryl aldehyde **2**, which was then subjected to a reductive amination by condensation of aldehyde **2** with *N*-Boc-cyclohexane-1,4-diamine, followed by reduction with NaBH(OAc)_3_ to afford secondary amine **3**. Selective alkylation of the newly generated secondary amine was achieved by treatment of amine **3** with NaH, followed by reaction with an alkylating reagent to give amine **4** in high yield. To complete the synthesis, amine **4** was reacted with acyl chloride in the presence of Et_3_N, and the formed amide was subjected to treatment with TFA to remove the Boc group.

### Substituent-modifications on the motif A

**Scheme 2:**
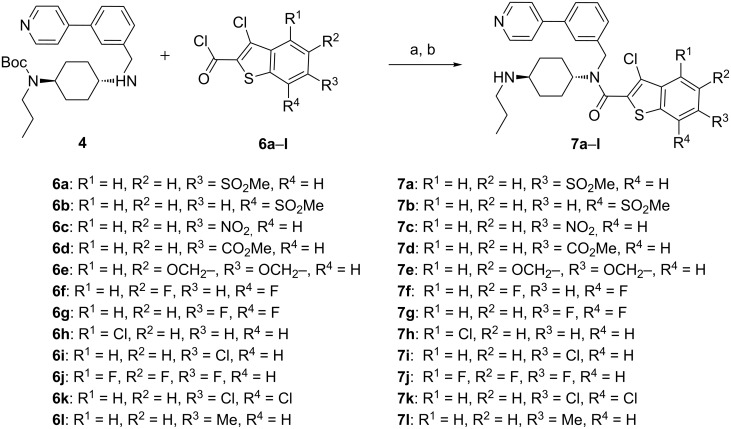
Substituent-modifications on the motif A. Reagents and conditions: (a) CH_2_Cl_2_, Et_3_N; (b) CH_2_Cl_2_, TFA.

**Scheme 3:**
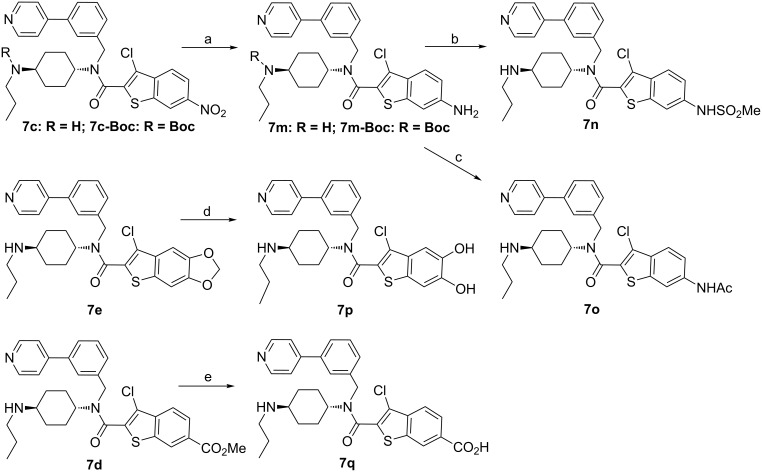
Substituent-modifications on the motif A. Reagents and conditions: (a) (i) FeCl_3_, Zn, H_2_O, DMF, 100 °C; (ii) TFA, CH_2_Cl_2_; (b) (i) MeSO_2_Cl, CH_2_Cl_2_, Et_3_N; (ii) TFA, CH_2_Cl_2_; (c) (i) AcCl, CH_2_Cl_2_, Et_3_N; (ii) TFA, CH_2_Cl_2_; (d) BBr_3_, CH_2_Cl_2_, −78 °C; (e) KOH, MeOH, H_2_O.

**Scheme 4:**
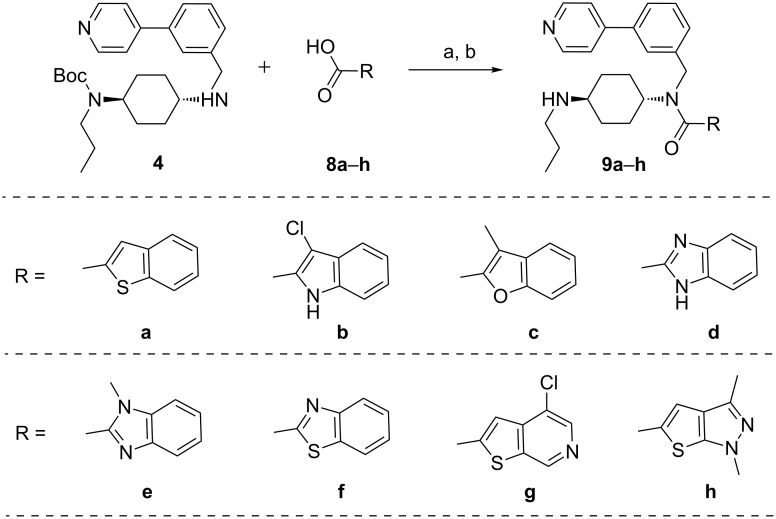
Core modification on the motif A. Reagents and conditions: (a) BOP, DIEA, DMF; (b) CH_2_Cl_2_, TFA.

The first series of derivatives is characterized by substituent modifications (Scheme 2, Scheme 3) and core modifications (Scheme 4) on the motif A. With respect to the substituent modifications, various groups, such as polar and hydrophobic groups, were introduced to the phenyl ring in motif A. Scheme 2 described the synthesis of derivatives **7a**–**l** through the reaction of compound **4** with a number of substituted acyl chlorides **6a**–**l**, which were prepared from the corresponding cinnamic acids by Higa cyclization [39–40]. It is noteworthy that some polar groups, including amino, hydroxy and sulfonamide, could not tolerate the conditions of Higa cyclization, and had to be introduced through transformation reactions after the N-acylation step.

As depicted in Scheme 3, NH_2_-derivative **7m** was prepared from the NO_2_-substituted precursor **7c** by the reduction of the nitro group to an amino group using an FeCl_3_–Zn system [41]. Subsequently, capping the amino group with a sulfonyl or acetyl group led to **7n** and **7o**, respectively. In regard to hydroxy or acid modification, the demethylation or hydrolysis released the corresponding hydroxy group or carboxylic acid. Accordingly, **7e** was treated with BBr_3_ in CH_2_Cl_2_ at −78 °C to afford the expected product **7p**. **7d** was directly subjected to basic hydrolysis in MeOH to yield carboxylic acid derivative **7q**.

To increase structural diversities, compound **4** was further reacted with various heteroaryl acids **8a**–**h** to yield **9a**–**h** (Scheme 4). The following considerations were taken for the selection of the heteroaryl acids: removal of the Cl atom (**8a**); replacement of the S atom with N or O atoms (**8b**, **8c**); two heteroatoms in a five-membered ring (**8d**–**f**); and a 5,5-fused ring heterocycle (**8h**), all of which will help us to more distinctly study the SAR in motif A. Compounds **8a**–**h** were either synthesized using reported methods [42–46] or are commercially available.

### Substituent-modifications on the motif B

**Figure 2:**
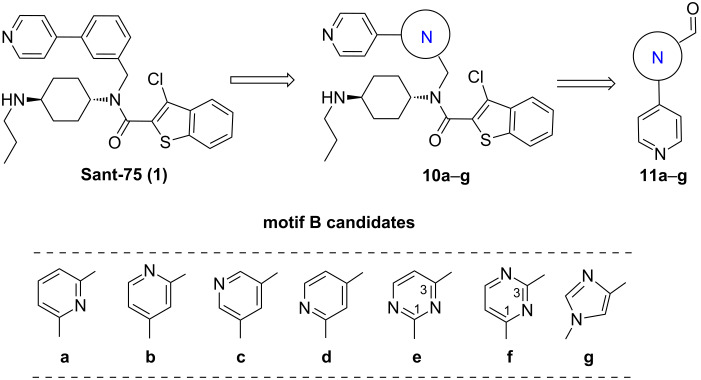
Core modification on the motif B.

**Scheme 5:**
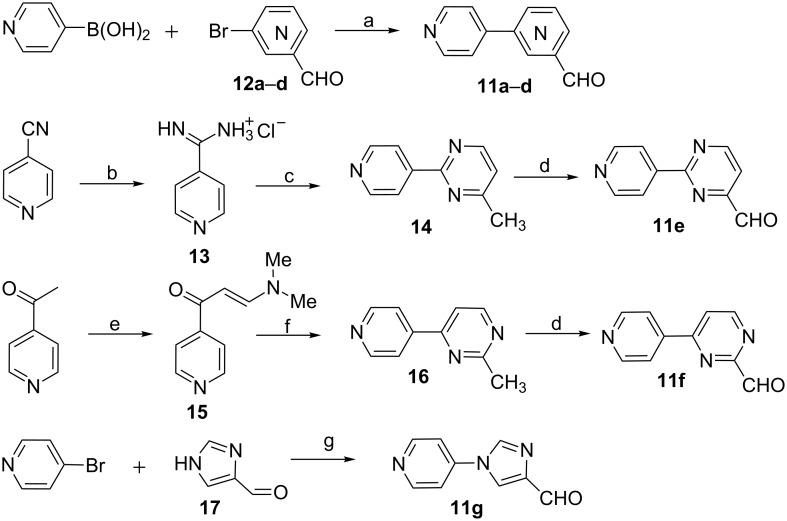
Synthesis of key intermediate biaryl aldehydes. Reagents and conditions: (a) Pd(OAc)_2_, PPh_3_, 1,4-dioxane, Na_2_CO_3_, 100 °C; (b) cat. NaOMe, MeOH; then NH_4_Cl; (c) acetylacetaldehyde dimethyl acetal, 1,4-dioxane, reﬂux; (d) SeO_2_, 1,4-dioxane, reflux; (e) DMF-DMA, PhMe, reflux; (f) acetamidine hydrochloride, Na, EtOH, reflux; (g) 1*H*-imidazole-4-carbaldehyde, DMF, CuI, Cs_2_CO_3_.

In the second series of novel scaffolds, structural modification was focused on alternatives to the phenyl ring to improve aqueous solubility. As illustrated in Figure 2, we replaced the phenyl ring with a variety of structurally diverse heteroaryl groups, including pyridine, pyrimidine and imidazole rings. In order to investigate the effect of the N-position in the heteroaryl ring, different pyridine and pyrimidine substituents were also incorporated. Generally, the compounds **10a**–**g** were synthesized following the general route in Scheme 1, by using aldehydes **11a**–**g** as key intermediates. As described in Scheme 5, **11a**–**d** were prepared through Pd-catalyzed Suzuki coupling of 4-pyridylboronic acid with corresponding formyl-functionalized pyridylbromides **12a**–**d**, which were commercially available. However, application of the reaction conditions identical to those used in the synthesis of compound **2** (Scheme 1) resulted in very low yields. After systematic optimization of the reaction conditions, we found that the reaction proceeded well in 1,4-dioxane at 100 °C for 8 h with Na_2_CO_3_ as base in the presence of Pd(OAc)_2_ as catalyst, and PPh_3_ as ligand, providing the products in 73–85% yield.

Different from the synthetic strategy of **11a**–**d**, the pyrimidine nucleus in biaryl aldehydes **11e**–**f** had to be constructed through condensation of a protected β-ketoaldehyde with the corresponding amidine. Compound **11e** was synthesized from isonicotinonitrile through three steps described previously. Isonicotinonitrile was converted to isonicotinamidine hydrochloride **13** upon reaction in MeOH in the presence of catalytic NaOMe, followed by treatment with NH_4_Cl. Then, condensation of **13** with acetylacetaldehyde dimethyl acetal in 1,4-dioxane afforded 6-methyl-2-(4-pyridinyl)pyrimidine **14** in 62% yield [47]. Consequent allylic oxidation of **14** with selenium dioxide gave **11e** in moderate yield. Likewise, compound **11f** was prepared though SeO_2_-oxidation of **16**, which was prepared by the reported method using 4-acetylpyridine as the starting material [48]. 4-Acetylpyridine was converted to enaminone intermediates **15** upon treatment with DMF-DMA in toluene under reflux, followed by condensation cyclization with acetamidine to give **16**. Compound **11g** could be installed in single step by Ullmann coupling of **17** with 4-bromopyridine, in good yield.

### Substituent-modifications on the motif C

**Scheme 6:**
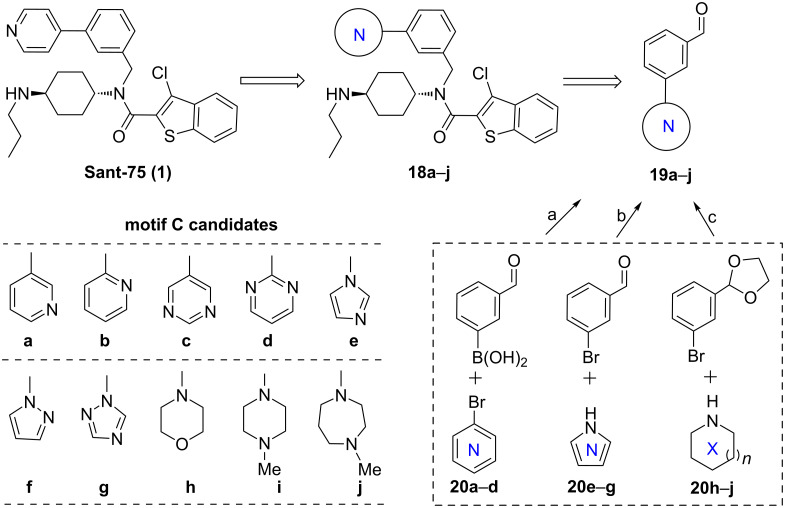
Chemical modifications on the motif C. Reagents and conditions: (a) Pd(OAc)_2_, PPh_3_, 1,4-dioxane, Na_2_CO_3_, 100 °C; (b) CuI, DMF, Cs_2_CO_3_, 120 °C; (c) (i) Pd_2_(dba)_3_, *rac*-BINAP, NaO*t*-Bu, PhMe, 100 °C; (ii) aq. HCl, THF.

In the third round of structural modifications, chemical diversity was explored by varying the nature of the heterocycles (motif C) and the position of the nitrogen atom in pyridine. As shown in Scheme 6, *para*-substituted pyridine was first replaced with *meta*- and *ortho*-pyridine, and other heterocycles, including pyrimidines and five-membered heteroaryl rings, as well as nonaromatic heterocycles, such as morpholine, piperazine and homo-piperazine, were also investigated. Accordingly, a small series of Sant-75 analogues **18a**–**j** were synthesized in a parallel fashion from the corresponding intermediates **19a**–**j**. For intermediates **19a**–**j**, different coupling reactions were required to achieve their synthesis, depending on the type of structure. **19a**–**d** were prepared through Suzuki coupling as described above, and **19e**–**g** were obtained in 70–85% yield by Ullmann coupling of 3-bromobenzaldehyde with an excess of the appropriate amines **20e**–**g** [49]. The Buchwald coupling of protected 3-bromobenzaldehyde with amines **20h**–**j** proceeded smoothly in toluene with NaO*t*-Bu as base by using a Pd_2_(dba)_3_/*rac*-BINAP catalysis system [50].

### Substituent-modifications on the motif D

**Scheme 7:**
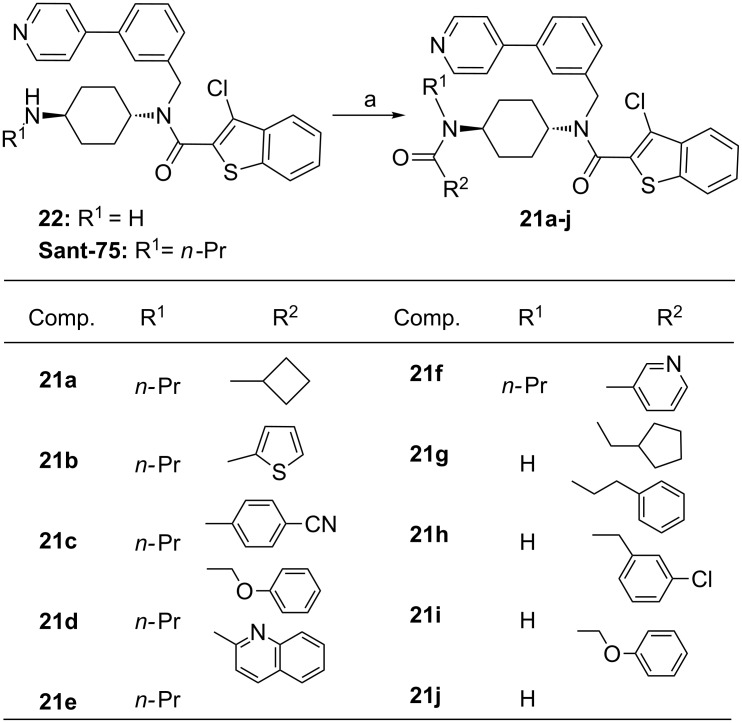
Chemical modifications on the motif D. Reagents and conditions: (a) R^2^COCl, CH_2_Cl_2_.

It has previously been shown that the substitution pattern on the nitrogen atom of *trans*-1,4-diaminocyclohexane moiety plays a critical role in the activity of the compounds as Hedgehog-pathway inhibitors. Therefore, the emphasis of the fourth series of structural optimization was placed on the nitrogen atom through capping of the amino group with various acyl chlorides, delivering the corresponding amides **21a**–**j**. It should be noted that the capping of the amino group can increase the metabolic stability of the compounds. The R^2^ group in modified compounds was extensively studied, including alkyl, cycloalkyl, phenyl and heteroaryl groups, and a selection of the generated compounds is listed in Scheme 7.

## Conclusion

By modification of our first generation of synthesis of **SAG** derivatives, a second generation of the synthetic program was established, which allows structurally diverse derivatives of Sant-75 to be synthesized in a systematic manner. The constructed library was fully characterized, which provides a foundation for the further investigation of their biological activities. The biological investigation of this library is currently underway in our laboratories, and the results will be reported in due course.

## Supporting Information

File 1 Experimental details.
