# Supplementary material for: Synthesis and characterization of Sant-75 derivatives as Hedgehog-pathway inhibitors
Source: Beilstein J Org Chem. 2012 Jun 6;8:841–9. doi: 10.3762/bjoc.8.94 (PMC3388872; doi:10.3762/bjoc.8.94)
Supplement: File 1 — Experimental details. [file Beilstein_J_Org_Chem-08-841-s001.pdf]

Supporting Information

for

Synthesis and characterization of Sant-75 derivatives as Hedgehog-pathway inhibitors

Chao Che[1,4], Song Li[1,4], Bo Yang[1], Shengchang Xin[1], Zhixiong Yu[1], Taofeng Shao[1], Chuanye Tao[1], Shuo Lin*[,1,3] and Zhen Yang*[,1,2]

[1]Laboratory of Chemical Genomics, School of Chemical Biology and Biotechnology, Peking University Shenzhen Graduate School, Shenzhen 518055, China; [2]Key Laboratory of Bioorganic Chemistry and Molecular Engineering of Ministry of Education and Beijing National Laboratory for Molecular Science (BNLMS), College of Chemistry, Peking University, Beijing 100871, China;[3]Department of Molecular, Cell and Developmental Biology, University of California Los Angeles, USA and [4]Shenzhen Shengjie Biotech Co., Ltd.

Email: Shou Lin* - shuolin@ucla.edu; Zhen Yang* - zyang@pku.edu.cn

* Corresponding author

Experimental details

General information:

Unless noted otherwise, all reactions were performed under a nitrogen atmosphere, and materials obtained from commercial suppliers were used without further purification. Purification of products was conducted by flash column chromatography on silica gel (200–300 mesh) purchased from Qing Dao Hai Yang Chemical Industry Co. ^{1}H NMR spectra were recorded on a Bruker 300 MHz or 500 MHz spectrometer using residual solvent (δ ($CDCl_3$) 7.26; δ (DMSO-d_6) 2.50) as the internal standard. All the coupling constants are reported in Hz. ^{13}C NMR spectra were recorded on the same instruments, and chemical shifts were measured relative to solvent resonances (δ ($CDCl_3$) = 77.0; δ (DMSO) = 39.5). Data for ^{1}H NMR are reported as follows: chemical shift (δ ppm), multiplicity (s = singlet, d = doublet, t = triplet, q = quartet, m = multiplet), integration and coupling constant (Hz), whereas ^{13}C NMR analyses were reported in terms of chemical shift. High-resolution mass spectra were obtained on a quadrupole time-of-flight (QTOF) mass spectrometer.

Abbreviations used:

Hh, Hedgehog; Ptch, Patched protein; Smo, Smoothened protein; SAR, structure–activity relationship; TEA, triethylamine; TFA, trifluoroacetic acid; THF, tetrahydrofuran; Boc, *tert*-butoxycarbonyl; BOP, benzotriazole-1-yl-oxy-tris-(dimethylamino)-phosphonium hexafluorophosphate; DIEA, *N*,*N*-diisopropylethyl-amine; DMF-DMA, *N*,*N*-dimethylformamide dimethyl acetal; PCC, pyridinium chlorochromate; BINAP, 2,2′-bis(diphenylphosphino)-1,1′-binaphthyl; DCM, dichloromethane; $Pd_2(dba)_3$, tris(dibenzylideneacetone)dipalladium(0); Ph, phenyl; DMF, *N*,*N*-dimethylformamide; HPLC, high-performance liquid chromatography; Ac, acetyl; rt, room temperature.

General procedure 1 (GP 1) – Reductive amination:

To a solution of bi-aryl aldehyde (1 mmol) in THF (20 mL) was added *trans*-*N*-Boc-1,4-diaminocyclohexane (1.2 mmol), and the mixture was stirred at room temperature for 30 min. To this solution was added $NaBH(AcO)_3$ (2.5 mmol) in portions at 0 °C, and the reaction mixture was stirred at room temperature for 4 h. The reaction was quenched by the addition of saturated aqueous Na_2CO_3 (5 mL), and the mixture was then extracted with chloroform (3 × 30 mL). The combined organic layers

were dried over anhydrous Na_2SO_4. The solvent was removed under vacuum, and the residue was purified by flash chromatography on silica gel (CH_2Cl_2/MeOH, 10/1) to give the desired product.

General procedure 2 (GP 2) – N-alkylation:

To a solution of the product from **GP 1** (1 mmol) in DMF (10 mL) was added NaH (3 mmol), and the reaction mixture was stirred at 0 °C for 1 h. To this solution was added 1-iodopropane (3 mmol), the resultant mixture was stirred at 0 °C for 0.5 h and for another 2h at room temperature. The reaction was quenched by the addition of a saturated solution of $NaHCO_3$ (15 mL), and extracted with CH_2Cl_2 (3 × 40 mL). The combined organic layers were washed with H_2O, brine and dried over Na_2SO_4. The solvent was removed under vacuum, and the residue was purified by flash chromatography on silica gel (CH_2Cl_2/MeOH, 10/1) to afford the *N*-propyl product.

General procedure 3 (GP 3) – Amidation using acyl chloride:

To a solution of *N*-propyl product in **GP 2** (0.2 mmol) and Et_3N (0.3 mmol) in CH_2Cl_2 (3 mL) was added acyl chloride (0.25 mmol), and the reaction mixture was stirred at room temperature for 2 h. The solvent was removed, and to the residue was added CH_2Cl_2 (2 mL) and trifluoroacetic acid (0.5 mL) at 0 °C, and the reaction mixture was stirred at room temperature for 1 h. The reaction was worked up by the addition of a saturated solution of Na_2CO_3 (2 mL) and then extracted with CH_2Cl_2 (3 × 10 mL), and the combined organic layers were finally dried over anhydrous Na_2SO_4. The solvent was removed under vacuum, and the residue was purified by flash chromatography on silica gel (CH_2Cl_2/acetone/TEA, 40/10/1) to give the target product.

General procedure 4 (GP 4) – Amidation using acids through BOP coupling:

To a solution of *N*-propyl product in **GP2** (0.2 mmol), acid (0.24 mmol) and BOP reagent (0.24 mmol) in DMF (4 mL) under nitrogen was added N,N-diisopropylethylamine (0.24 mmol). The reaction mixture was stirred at room temperature for 12 h. The solvent was removed under vacuum, and to the residue was added CH_2Cl_2 (2 mL) and trifluoroacetic acid (0.5 mL) at 0 °C, and the reaction mixture was stirred at room temperature for 1 h. The reaction was worked up by the addition of a saturated solution of Na_2CO_3 (2 mL) and then extracted with CH_2Cl_2 (3 × 10 mL), and the combined organic layers were finally dried over anhydrous Na_2SO_4. The solvent was removed under vacuum, and the residue was purified by flash

chromatography on silica gel (CH_2Cl_2/acetone/TEA, 40/10/1) to give the target product.

Characterization data of the synthesized compounds:

Compound 7a (GP 3); yield (56%) starting from **4** and **6a**; yellow solid; ^{1}H NMR (300 MHz, $CDCl_3$) δ 8.66 (d, *J* = 4.8 Hz, 2H), 8.50 (s, 1H), 8.05 (s, 2H), 7.35–7.70 (m, 6H), 4.84 (s, 2H), 3.68–3.72 (m, 1H), 3.14 (s, 3H), 2.31–2.56 (m, 3H), 1.25–1.94 (m, 10H), 0.83 (t, *J* = 7.0 Hz, 3H); HRMS (*m/z*): [M^+] calcd for $C_{31}H_{35}ClN_3O_3S_2$, 596.1808; found, 596.1810.

Compound 7b (GP 3); yield (61%) starting from **4** and **6b**; yellow solid; ^{1}H NMR (300 MHz, $CDCl_3$) δ 8.66 (d, *J* = 4.8 Hz, 2H), 7.92–8.26 (br, m, 2H), 7.27–7.69 (m, 7H), 4.83 (s, 2H), 3.84 (br, *m*, 1H), 3.15 (s, 3H), 2.62–2.83 (m, 3H), 1.25–2.21 (m, 10H), 0.88 (t, *J* = 6.8 Hz, 3H); HRMS (*m/z*): [M^+] calcd for $C_{31}H_{35}ClN_3O_3S_2$, 596.1808; found, 596.1809.

Compound 7c (GP 3); yield (69%) starting from **4** and **6c**; yellow solid; ^{1}H NMR (300 MHz, $CDCl_3$) δ 8.68 (d, *J* = 5.4 Hz, 2H), 8.28–8.42 (br, 1H), 7.90–8.11 (br, 1H), 7.26–7.67 (m, 7H), 4.63–4.82 (br, 2H), 3.78–4.18 (br, 1H), 2.68–2.90 (br, m, 3H), 1.25–2.21 (m, 10H), 0.89 (t, *J* = 7.2 Hz, 3H); HRMS (*m/z*): [M^+] calcd for $C_{30}H_{32}ClN_4O_3S$, 563.1884; found, 563.1898.

Compound 7d (GP 3); yield (32%) starting from **4** and **6d**; yellow solid; ^{1}H NMR (300 MHz, $CDCl_3$) δ 8.57–8.66 (m, 3H), 7.26–8.19 (m, 8H), 4.84 (s, 2H), 3.99 (s, 3H), 3.76 (m, 1H), 2.35–2.60 (m, 3H), 1.28–2.04 (m, 10H), 0.83 (t, *J* = 6.7 Hz, 3H); HRMS (*m/z*): [M^+] calcd for $C_{32}H_{35}ClN_3O_3S$, 576.2088; found, 576.2079.

Compound 7e (GP 3); yield (72%) starting from **4** and **6e**; yellow solid; ^{1}H NMR (300 MHz, $CDCl_3$) δ 8.65 (d, *J* = 4.5 Hz, 2H), 7.30–7.68 (m, 6H), 7.15–7.20 (m, 2H), 6.07 (s, 2H), 4.77 (s, 2H), 3.85 (m, 1H), 2.33–2.55 (m, 3H), 1.83–1.97 (m, 5H), 1.26 (m, 5H), 0.87 (t, *J* = 6.7 Hz, 3H); HRMS (*m/z*): [M^+] calcd for $C_{31}H_{33}ClN_3O_3S$ [M^+]: 562.1931; found 562.1979.

Compound 7f (GP 3); yield (75%) starting from **4** and **6f**; yellow solid; ^{1}H NMR (300 MHz, $CDCl_3$) δ 8.68 (d, *J* = 6.0 Hz, 2H), 7.26–7.66 (m, 7H), 7.02 (br, 1H), 4.63–4.81 (br, 2H), 3.74–3.79 (*m*, 1H), 2.63–2.78 (br, m, 3H), 1.25–2.19 (m, 10H), 0.89 (t, *J* = 7.2 Hz, 3H); HRMS (*m/z*): [M^+] calcd for $C_{30}H_{31}ClF_2N_3OS$, 554.1844, found, 554.1848.

Compound 7g (GP 3); yield (67%) starting from **4** and **6g**; yellow solid; ^{1}H NMR (300 MHz, $CDCl_3$) δ 8.68 (d, *J* = 4.5 Hz, 2H), 7.32–7.61 (m, 8H), 4.65–4.79 (br, 2H), 3.83–4.14 (br, 1H), 2.64–2.84 (br, *m*, 3H), 1.25–2.21 (m, 10H), 0.90 (t, *J* = 7.2 Hz, 3H); HRMS (*m/z*): [M$^+$] calcd for $C_{30}H_{31}ClF_2N_3OS$, 554.1844, found, 554.1825.

Compound 7h (GP 3); yield (77%) starting from **4** and **6h**; yellow solid; ^{1}H NMR (300 MHz, $CDCl_3$) δ 8.66 (s, 2H), 7.26–7.71 (m, 9H), 4.63 (s, 2H), 3.77 (m, 1H), 2.60–2.74 (m, 3H), 1.82–1.98 (m, 4H), 1.26–1.68 (m, 6H), 0.87 (t, *J* = 6.7 Hz, 3H); HRMS (*m/z*): [M$^+$] calcd for $C_{30}H_{32}Cl_2N_3OS$, 552.1643; found, 552.1625.

Compound 7i (GP 3); yield (82%) starting from **4** and **6i**; yellow solid; ^{1}H NMR (300 MHz, $CDCl_3$) δ 8.66 (d, *J* = 4.2 Hz, 2H), 7.45–7.82 (m, 9H), 4.81 (s, 2H), 3.77 (m, 1H), 2.34–2.47 (m, 3H), 1.78–1.94 (m, 4H), 1.24–1.68 (m, 6H), 0.87 (t, *J* = 6.7 Hz, 3H); HRMS (*m/z*): [M$^+$] calcd for $C_{30}H_{32}Cl_2N_3OS$, 552.1643; found, 552.1636.

Compound 7j (GP 3); yield (74%) starting from **4** and **6j**; yellow solid; ^{1}H NMR (300 MHz, $CDCl_3$) δ 8.67 (d, *J* = 6.0 Hz, 2H), 7.30–7.65 (m, 7H), 4.63–4.79 (br, 2H), 3.78–4.19 (br, 1H), 2.63–2.84 (br, m, 3H), 1.25–2.17 (m, 10H), 0.88 (t, *J* = 7.2 Hz, 3H); HRMS (*m/z*): [M$^+$] calcd for $C_{30}H_{30}ClF_3N_3OS$, 572.1750; found, 572.1747.

Compound 7k (GP 3); yield (71%) starting from **4** and **6k**; yellow solid; ^{1}H NMR (300 MHz, $CDCl_3$) δ 8.66 (d, *J* = 4.2 Hz, 2H), 7.46–7.68 (m, 8H), 4.69–4.85 (br, 2H), 3.79 (m, 1H), 2.42–2.72 (m, 3H), 1.80–2.08 (m, 4H), 1.24–1.68 (m, 6H), 0.87 (t, *J* = 6.7 Hz, 3H); HRMS (*m/z*): [M$^+$] calcd for $C_{30}H_{31}Cl_3N_3OS$, 586.1253; found, 586.1232.

Compound 7l (GP 3); yield (65%) starting from **4** and **6l**; yellow solid; ^{1}H NMR (300 MHz, $CDCl_3$) δ 8.65 (d, *J* = 4.5 Hz, 2H), 7.31–7.72 (m, 9H), 4.69 (br, 2H), 3.88–4.12 (br, 1H), 2.76–2.85 (m, 3H), 2.48 (s, 3H), 1.92–2.10 (m, 4H), 1.26–1.61 (m, 6H), 0.87 (t, *J* = 6.7 Hz, 3H); HRMS (*m/z*): [M$^+$] calcd for $C_{31}H_{35}ClN_3OS$, 532.2189; found, 532.2190.

Compound 7m. To a mixture of **7c-Boc** (331mg, 0.5 mmol) in a mixture of DMF and water (1:1, 5 mL) was added $FeCl_3$ (225 mg, 1.5 mmole) and zinc dust (325 mg, 5 mmole), and the mixture was heated at 100 °C for 1 h. When the reaction was complete, the mixture was filtered and filtrate was diluted with water (10 mL) and basified by adding a saturated solution of Na_2CO_3. The solution was then extracted with CH_2Cl_2 (3 × 10 mL) and dried over anhydrous Na_2SO_4. After concentration under vacuum, the residue was treated with trifluoroacetic acid (2 mL) in 10 mL of CH_2Cl_2 at 0 °C, and the reaction mixture was stirred at room temperature for 1 h. The reaction

was worked up by the addition of a saturated solution of Na_2CO_3 (10 mL) and then extracted with CH_2Cl_2 (3 × 30 mL), and the combined organic layers were finally dried over anhydrous Na_2SO_4. The solvent was removed under vacuum, and the residue was purified by a flash chromatography on silica gel (DCM/MeOH, 30/1) to give **7m** (191 mg, 72%). Yellow solid; ^{1}H NMR (300 MHz, DMSO) δ 8.63 (*s,* 2H), 7.47–7.64 (m, 7H), 6.83–7.02 (m, 2H), 5.71 (s, 2H), 4.75 (*s*, 2H), 3.72 (m, 1H), 2.70–2.89 (m, 3H), 1.21–2.07 (m, 10H), 0.87 (t, *J* = 6.7 Hz, 3H); HRMS (*m/z*): [M^+] calcd for $C_{30}H_{34}ClN_4OS$, 533.2142; found, 533.2149.

Compound 7n. To a solution of **7m-Boc** (63 mg, 0.1mmol) and Et_3N (20.8 μL, 0.15 mmol) in 10 mL of dry CH_2Cl_2 was added $MeSO_2Cl$ (14 mg, 0.12 mmol), and the reaction mixture was stirred at room temperature for 4 h. The solvent was removed, and the residue was treated with trifluoroacetic acid (0.5 mL) in 2 mL of CH_2Cl_2 at 0 °C, and the reaction mixture was stirred at room temperature for 1 h. The reaction was worked up by the addition of a saturated solution of Na_2CO_3 (2 mL) and then extracted with CH_2Cl_2 (3 × 10 mL), and the combined organic layers were finally dried over anhydrous Na_2SO_4. The solvent was removed under vacuum, and the residue was purified by a flash chromatography on silica gel (DCM/MeOH, 40/1) to give product **7n** (48 mg, 78%). Yellow solid; ^{1}H NMR (300 MHz, DMSO) δ 8.64 (s*,* 2H), 7.29–7.80 (m, 9H), 4.79 (s, 2H), 2.98 (s, 3H), 2.35–2.50 (m, 3H), 1.21–2.07 (m, 10H), 0.87 (t, *J* = 6.7 Hz, 3H); HRMS (*m/z*): [M^+] calcd for $C_{31}H_{36}ClN_4O_3S_2$, 611.1917; found, 611.1922.

Compound 7o. To a solution of **7m-Boc** (63 mg, 0.1mmol) and Et_3N (20.8 μL, 0.15 mmol) in CH_2Cl_2 (5 mL) was added acetyl chloride (10 mg, 0.12 mmol), and the reaction mixture was stirred at room temperature for 3 h. The solvent was removed, and the residue was treated with trifluoroacetic acid (0.5 mL) in 2 mL of CH_2Cl_2 at 0 °C, and the reaction mixture was stirred at room temperature for 1 h. The reaction was worked up by addition of a saturated solution of Na_2CO_3 (2 mL) and then extracted with CH_2Cl_2 (3 × 10 mL), and the combined organic layers were finally dried over anhydrous Na_2SO_4. The solvent was removed under vacuum, and the residue was purified by flash chromatography on silica gel (CH_2Cl_2/MeOH, 30/1) to give the product **7o** (42 mg, 74%). Yellow solid; ^{1}H NMR (300 MHz, $CDCl_3$) δ 8.65 (s, 2H), 8.25 (s, 1H), 7.28–7.65 (m, 9H), 4.78 (br, 2H), 3.88–4.20 (br, 1H), 2.70–2.88 (m, 3H), 2.18 (s, 3H), 1.92–2.10 (m, 4H), 1.26–2.14 (m, 10H), 0.87 (t, *J* = 6.7 Hz, 3H); HRMS (*m/z*): [M^+] calcd for $C_{32}H_{36}ClN_4O_2S$, 575.2247; found, 575.2246.

Compound 7p. To a solution of **7e** (56 mg, 0.1 mmol) in dry CH_2Cl_2 (5 mL) was added BBr_3 (22.6 μL, 0.24 mmol) under nitrogen through a syringe at −78 °C, and the reaction mixture was allowed to warm up to room temperature, and continuously stirred overnight. The reaction was worked up by the addition of ethyl acetate (30 mL), and then the mixture was washed sequentially with brine (3 × 4 mL) and water (2 × 4 mL), and the organic layer was dried over Na_2SO_4. The solvent was removed under vacuum, and the residue was purified by a flash chromatography (CH_2Cl_2/MeOH, 20/1) to give **7p** (37 mg) in 68% yield. Yellow solid; ^{1}H NMR (300 MHz, DMSO) δ 9.79 (br, 2H), 8.79–8.95 (m, 3H), 7.18–8.26 (m, 7H), 7.15–7.20 (m, 2H), 4.80 (s, 2H), 2.92–2.98 (m, 1H), 2.65–2.75 (m, 2H), 2.02–2.15 (m, 2H), 1.56–1.78 (m, 6H), 1.20 (m, 2H), 0.87 (t, J = 6.7 Hz, 3H); HRMS (*m/z*): [M^+] calcd for $C_{30}H_{33}ClN_3O_3S$, 550.1931; found, 550.1947.

Compound 7q. A mixture of **7d** (28 mg, 0.05 mmol) in 2 mL of methanol and 2 mL of aqueous 0.25 N KOH was stirred at 40 °C for 2 h. The mixture was stirred into 5 mL of water, and water was added until a solution was obtained. The aqueous solution was extracted with ether (2 × 4 mL) to remove the impurity, and then acidified with concentrated aqueous HCl, filtered, rinsed with water, and dried to afford 18 mg (63%) of **7q**. Yellow solid; ^{1}H NMR (300 MHz, DMSO) δ 8.58–8.66 (m, 3H), 7.46–8.14 (m, 8H), 4.83 (s, 2H), 4.29 (m, 1H), 2.66–2.91 (m, 3H), 1.53–2.07 (m, 6H), 1.15–1.21 (m, 4H), 0.83 (t, J = 6.7 Hz, 3H); HRMS (*m/z*): [M+] calcd for $C_{31}H_{33}ClN_3O_3S$, 562.1931; found, 562.1935.

Compound 9a (GP 4); yield (37%) starting from **4** and **8a**; yellow solid; ^{1}H NMR (400 MHz, $CDCl_3$) δ 8.66 (dd, J = 1.2, 4.5 Hz, 2H), 7.83 (d, J = 7.2 Hz, 1H), 7.81 (s, br, 1H), 7.34–7.57 (m, 9H), 4.84 (s, 2H), 4.23 (m, 1H), 2.64–2.77 (m, 3H), 1.41–2.12 (m, 10H), 0.88 (t, J = 7.56 Hz, 3H); HRMS (*m/z*): [M^+] calcd for $C_{30}H_{34}N_3OS$, 484.2423, found, 484.2431.

Compound 9b (GP 4); yield (44%) starting from **4** and **8b**; yellow solid; ^{1}H NMR (300 MHz, $CDCl_3$) δ 8.62 (d, J = 4.5 Hz, 2H), 7.14–7.61(m, 10H), 4.75 (s, 2H), 4.25 (m, 1H), 2.56–2.59 (m, 3H), 1.44–2.02 (m, 10H), 0.87 (t, J = 7.5 Hz, 3H); HRMS (*m/z*): [M^+] calcd for $C_{30}H_{34}N_4OCl$, 501.2421; found, 501.2403.

Compound 9c (GP 4); yield (47%) starting from **4** and **8c**; yellow solid; ^{1}H NMR (300 MHz, $CDCl_3$) δ 8.66 (d, J = 4.2 Hz, 2H), 7.26–7.52 (s, 10H), 4.75 (s, 2H), 4.00–4.15 (br, 1H), 2.78–2.90 (m, 3H), 2.44 (*s*, 3H), 2.22–2.25 (m, 2H), 1.64–1.98 (m, 8H), 0.92

(t, J = 7.5 Hz, 3H); HRMS (*m/z*): [M^+] calcd for $C_{31}H_{36}N_3O_2$, 482.2808; found, 482.2811.

Compound 9d (GP 4); yield (37%) starting from **4** and **8d**; yellow solid; ^{1}H NMR (300 MHz, $CDCl_3$) δ 8.62 (d, J = 4.8 Hz, 2H), 7.26–7.56 (s, 10H), 6.15 (br, 0.55H), 5.74 (s, 0.9H), 4.87 (s, 1.1H), 4.38 (br, 0.45H), 2.54–2.67 (m, 4H), 1.54–2.09 (m, 10H), 0.92 (t, J = 7.5 Hz, 3H); HRMS (*m/z*): [M+] calcd for $C_{29}H_{34}N_5O$, 468.2763, found, 468.2765.

Compound 9e (GP 4); yield (33%) starting from **4** and **8e**; yellow solid; ^{1}H NMR (300 MHz, $CDCl_3$) δ 8.55–8.66 (m, 2H), 7.10–7.79 (m, 10H), 4.84–4.97 (m, 2H), 4.41–4.71 (br, 1H), 3.59 (s, 3H), 2.83–2.89 (m, 3H), 1.60–2.32 (m, 10H), 0.92 (t, J = 7.5 Hz, 3H); HRMS (*m/z*): [M^+] calcd for $C_{30}H_{36}N_5O$, 482.2920; found, 482.2906.

Compound 9f (GP 4); yield (41%) starting from **4** and **8f**; yellow solid; ^{1}H NMR (300 MHz, $CDCl_3$) δ 8.64 (s, 2H), 7.36–8.16 (m, 10H), 5.50 (br, 0.45H), 5.40 (s, 1.1H), 4.85 (s, 0.8 H), 4.13 (br, 0.55H), 2.80–2.91(m, 3H), 2.10–2.28 (m, 2H), 1.62–2.10 (m, 8H), 0.96 (t, J = 7.5 Hz, 3H); HRMS (*m/z*): [M^+] calcd for $C_{29}H_{33}N_4OS$, 485.2375; found, 485.2368.

Compound 9g (GP 4); yield (28%) starting from **4** and **8g**; yellow solid; ^{1}H NMR (300 MHz, $CDCl_3$) δ 9.01 (s, 1H), 8.71 (m, 2H), 8.49 (s, 1H), 7.28–7.62 (m,7H), 4.84 (s, 2H), 4.12–4.15 (m, 1H), 2.82–2.85 (m, 3H), 1.71–2.23 (m, 10H), 0.96 (t, J = 7.5 Hz, 3H); HRMS (*m/z*): [M^+] calcd for $C_{29}H_{32}N_4OSCl$, 519.1990; found, 519.1985.

Compound 9h (GP 4); yield (30%) starting from **4** and **8h**; yellow solid; ^{1}H NMR (300 MHz, $CDCl_3$) δ 8.68 (s, 2H), 7.40–7.51 (m, 6H), 7.08 (s, 1H), 4.87 (s, 2H), 4.21 (m, 1H), 3.88 (s, 3H), 2.72–2.95 (m, 3H), 2.32 (s, 3H), 1.75–2.24 (m, 10H), 0.93 (t, J = 7.5 Hz, 3H); HRMS (*m/z*) calcd for $C_{29}H_{36}N_5OS$, 502.2641; found, 502.2629.

General Procedure 5 (GP 5):

To an oven-dried round-bottomed flask with a side-arm and condenser, was added bromopyridine **12** (185 mg, 1.0 mmol), 4-pyridylboronic acid (135 mg, 1.1 mmol), and anhydrous 1,4-dioxane (5 mL) under N_2. A degassed aqueous solution of Na_2CO_3 (2 M, 1 mL) was then added via syringe to the vigorously stirred reaction mixture, followed by the addition of $Pd(OAc)_2$ (11.2 mg, 0.05 mmol) and PPh_3 (65.5 mg, 0.25 mmol). The reaction flask was then purged by using three N_2/vacuum back-fill cycles and the contents were then stirred at 95 °C for 10 h. After cooling to room temperature, the reaction mixture was then poured into 5 mL of water and then

extracted with CH_2Cl_2. The organic layer was washed successively with water, brine and dried over anhydrous Na_2SO_4. The solvent was removed under vacuum, and the residue was purified by flash chromatography (*n*-hexane/ethyl acetate, 4/1) to give the desired product **11**.

Compound 11a (GP 5); yield (73%) starting from 4-pyridylboronic acid and **12a**; white solid; ^{1}H NMR (400 MHz, $CDCl_3$) δ 10.17 (s, 1H), 8.74 (d, *J* = 5.2 Hz, 2H), 7.99–8.04 (m, 5H); ^{13}C NMR (100 MHz, $CDCl_3$) δ 193.36, 155.13, 153.01, 150.66, 145.10, 138.30, 124.72, 121.36, 121.04.

Compound 11b (GP 5); yield (82%) starting from 4-pyridylboronic acid and **12b**; white solid; ^{1}H NMR (400 MHz, $CDCl_3$) δ 10.17 (s, 1H), 8.91 (d, *J* = 5.2 Hz, 1H), 8.78 (d, *J* = 5.2 Hz, 2H), 8.23 (s, 1H), 7.78 (d, *J* =3.6 Hz, 1H), 7.59 (d, *J* = 5.6 Hz, 1H); ^{13}C NMR (100 MHz, $CDCl_3$) δ 193.08, 153.71, 151.10, 150.86, 146.94, 144.47, 125.33, 121.39, 119.27.

Compound 11c (GP 5); yield (85%) starting from 4-pyridylboronic acid and **12c**; white solid; ^{1}H NMR (400 MHz, $CDCl_3$) δ 10.23 (*s*, 1H), 9.13 (dd, *J* = 2.4, 6.4 Hz, 2H), 8.78 (d, *J* = 6.0 Hz, 2H), 8.41 (s, 1H), 7.56 (d, *J* = 6.0 Hz, 2H); ^{13}C NMR (100 MHz, $CDCl_3$) δ 190.22, 152.95, 152.23, 150.82, 143.79, 134.58, 133.73, 131.48, 121.58.

Compound 11d (GP 5); yield (79%) starting from 4-pyridylboronic acid and **12d**; white solid; ^{1}H NMR (400 MHz, $CDCl_3$) δ 10.23 (s, 1H), 9.13 (dd, *J* = 2.0, 8.8 Hz, 2H), 8.78 (d, *J* = 6.0 Hz, 2H), 8.41 (s, 1H), 7.56 (d, *J* = 5.6 Hz, 2H); ^{13}C NMR (100 MHz, $CDCl_3$) δ 190.21, 152.95, 152.23, 150.82, 143.79, 134.59, 133.73, 131.49, 121.58.

Compound 10a (GP 1, GP 2, GP 3); yield (41%) starting from **11a**; yellow solid; ^{1}H NMR (300 MHz, $CDCl_3$) δ 8.75 (d, *J* = 5.7 Hz, 2H), 7.39–7.94 (m, 9H), 4.76–4.93 (br, 2H), 4.52 (br, 0.4H), 3.82 (br, 0.6H), 2.52–2.66 (m, 3H), 1.43–2.33 (m, 10H), 0.88 (t, *J* = 7.2 Hz, 3H). HRMS (*m/z*): [M^+] calcd for $C_{29}H_{32}ClN_4OS$, 519.1985; found, 519.1986.

Compound 10b (GP 1, GP 2, GP 3); yield (36%) starting from **11b**; yellow solid; ^{1}H NMR (300 MHz, $CDCl_3$) δ 8.65–8.75 (m, 3H), 7.41–7.78 (m, 8H), 4.74–4.93 (br, 2H), 4.42 (m, 0.4H), 3.85 (m, 0.6H), 2.68–2.86 (m, 3H), 1.71–2.20 (m, 10H), 0.88 (t, *J* = 7.2 Hz, 3H). HRMS (*m/z*): [M^+] calcd for $C_{29}H_{32}ClN_4OS$, 519.1985; found, 519.1976.

Compound 10c (GP 1, GP 2, GP 3); yield (34%) starting from **11c**; yellow solid; ^{1}H NMR (300 MHz, $CDCl_3$) δ 8.64–8.80 (m, 4H), 7.38–7.88 (m, 7H), 4.72–4.78 (m, 2H),

3.82–3.92 (m, 1H), 2.86–2.83 (m, 3H), 1.62–2.14 (m, 10H), 0.88 (t, J = 7.2 Hz, 3H); HRMS (*m/z*): [M^+] calcd for $C_{29}H_{32}N_4OSCl$, 519.1985; found, 519.1991.

Compound 10d (GP 1, GP 2, GP 3); yield (29%) starting from **11d**; yellow solid; ^{1}H NMR (300 MHz, $CDCl_3$) δ 9.22 (br, 1H), 8.70–8.76 (m, 2H), 7.26–7.89 (m, 8H), 4.77 (s, 2H), 3.86–3.92 (m, 1H), 2.68–2.89 (m, 3H), 1.67–2.16 (m, 10H), 0.88 (t, J = 7.2 Hz, 3H); HRMS (*m/z*): [M^+] calcd for $C_{29}H_{32}N_4OSCl$, 519.1985; found, 519.1982.

Compound 10e (GP 1, GP 2, GP 3); yield (35%) starting from **11e**; yellow solid; ^{1}H NMR (300 MHz, $CDCl_3$) δ 8.78–8.83 (m, 3H), 7.46–7.86 (m, 7H), 4.82 (br, 2H), 4.52 (m, 0.4H), 3.92 (m, 0.6H), 2.73–2.90 (m, 3H), 1.66–2.21 (m, 10H), 0.88 (t, J = 7.2 Hz, 3H); HRMS (*m/z*): [M^+] calcd for $C_{28}H_{31}ClN_5OS$, 520.1938; found, 520.1943.

Compound 10f (GP 1, GP 2, GP 3); yield (40%) starting from **11f**; yellow solid; ^{1}H NMR (300 MHz, $CDCl_3$) δ 8.77–8.84 (m, 3H), 7.39–8.04 (m, 7H), 4.84–4.98 (m, 2H), 3.96 (m, 1H), 2.74–2.92 (m, 3H), 1.62–2.22 (m, 10H), 0.94 (t, J = 7.4Hz, 3H); HRMS (*m/z*): [M^+] calcd for $C_{28}H_{31}ClN_5OS$, 520.1938; found, 520.1924.

Compound 10g (GP 1, GP 2, GP 3); yield (37%) starting from **11a**; yellow solid; ^{1}H NMR (300 MHz, $CDCl_3$) δ 8.67 (s, 2H), 7.16–7.95 (m, 8H), 4.64 (br, 2H), 3.70–3.76 (m, 1H), 2.77–2.96 (m, 3H), 1.40–2.16 (m, 10H), 0.88 (t, J = 7.2Hz, 3H); HRMS (*m/z*): [M^+] calcd for $C_{27}H_{31}ClN_5OS$, 508.1938; found, 508.1946.

Compound 18a (GP 1, GP 2, GP 3); yield (48%) starting from **19a**; yellow solid; ^{1}H NMR (300 MHz, $CDCl_3$) δ 9.21 (br, 2H), 8.77–8.86 (m, 1H), 8.62 (s, 1H), 7.42–7.86 (m, 8H), 4.66–4.81 (br, 2H), 3.87–4.16 (m, 1H), 2.72–2.88 (m, 3H), 1.68–2.17 (m, 10H), 0.87 (t, J = 7.2 Hz, 3H); HRMS (*m/z*): [M^+] calcd for $C_{30}H_{31}ClN_3OS$, 518.2033; found, 518.2033.

Compound 18b (GP 1, GP 2, GP 3); yield (45%) starting from **19b**; yellow solid; ^{1}H NMR (300 MHz, $CDCl_3$) δ 8.70 (br, 1H), 7.28–7.86 (m, 11H), 4.68–4.84 (br, 2H), 3.84–4.15 (m, 1H), 2.72–2.92 (m, 3H), 1.66–2.06 (m, 10H), 0.87 (t, J = 7.2 Hz, 3H); HRMS (*m/z*): [M^+] calcd for $C_{30}H_{31}ClN_3OS$, 518.2033; found, 518.2030.

Compound 18c (GP 1, GP 2, GP 3); yield (36%) starting from **19c**; yellow solid; ^{1}H NMR (300 MHz, $CDCl_3$) δ 9.21 (s, 1H), 8.79–8.98 (br, 2H), 7.49–7.84 (m, 8H), 4.68–4.82 (br, 2H), 3.80–4.28 (m, 1H), 2.45–2.62 (m, 3H), 1.25–2.06 (m, 10H), 0.87 (t, J = 7.2 Hz, 3H); HRMS (*m/z*): [M^+] calcd for $C_{29}H_{32}ClN_4OS$, 519.1985; found, 519.1981.

Compound 18d (GP 1, GP 2, GP 3); yield (41%) starting from **19d**; yellow solid; ^{1}H NMR (300 MHz, $CDCl_3$) δ 8.82 (d, *J* = 4.5 Hz, 2H), 8.26–8.58 (m, 2H), 7.62–7.91 (m, 2H), 7.43–7.58 (m, 4H), 7.22 (t, *J* = 6.9 Hz, 1H), 4.68–4.85 (br, 2H), 3.87–4.16 (m, 1H), 2.65–2.87 (m, 3H), 1.60–2.19 (m, 10H), 0.87 (t, *J* = 7.2 Hz, 3H); HRMS (*m/z*): [M^+]calcd for $C_{29}H_{32}ClN_4OS$, 519.1985; found, 519.1989.

Compound 18e (GP 1, GP 2, GP 3); yield (32%) starting from **19e**; yellow solid; ^{1}H NMR (300 MHz, $CDCl_3$) δ 7.71–7.89 (m, 3H), 7.19–7.49 (m, 8H), 4.73 (br, 2H), 3.89–4.15 (m, 1H), 2.67–2.89 (m, 3H), 1.62–2.19 (m, 10H), 0.87 (t, *J* = 7.2 Hz, 3H); HRMS (*m/z*): [M^+] calcd for $C_{28}H_{32}ClN_4OS$, 507.1985; found, 507.1978.

Compound 18f (GP 1, GP 2, GP 3); yield (35%) starting from **19f**; yellow solid; ^{1}H NMR (300 MHz, $CDCl_3$) δ 7.16–7.85 (m, 10H), 6.47 (s, 1H), 4.65–4.79 (br, 2H), 3.84–4.16 (m, 1H), 2.67–2.89 (m, 3H), 1.68–2.18 (m, 10H), 0.88 (t, *J* = 7.2 Hz, 3H); HRMS (*m/z*): [M^+] calcd for $C_{28}H_{32}ClN_4OS$, 507.1985; found, 507.1989.

Compound 18g (GP 1, GP 2, GP 3); yield (27%) starting from **19g**; yellow solid; ^{1}H NMR (300 MHz, $CDCl_3$) δ 8.50–8.62 (m, 2H), 8.09 (s, 1H), 7.48–7.84 (m, 8H), 4.67–4.78 (br, 2H), 3.86–4.21 (m, 1H), 2.72–2.86 (m, 3H), 1.73–2.19 (m, 10H), 0.87 (t, *J* = 7.2 Hz, 3H); HRMS (*m/z*): [M^+] calcd for $C_{27}H_{31}ClN_5OS$, 508.1938; found, 508.1932.

Compound 18h (GP 1, GP 2, GP 3); yield (43%) starting from **19h**; yellow solid; ^{1}H NMR (300 MHz, $CDCl_3$) δ 7.78–7.81 (m, 2H), 7.43–7.52 (m, 2H), 7.00–7.22 (m, 1H) 6.71–6.79 (m, 3H), 4.55–4.68 (m, 2H), 3.83–4.10 (m, 1H), 3.83 (s, 4H), 3.21–3.25 (m, 4H), 2.68–2.86 (m, 3H), 2.57–2.60 (br, 4H), 1.79–2.23 (m, 10H), 0.91 (t, *J* = 7.2 Hz, 3H); HRMS (*m/z*): [M^+] calcd for $C_{29}H_{37}ClN_3O_2S$, 526.2295; found, 526.2296.

Compound 18i (GP 1, GP 2, GP 3); yield (38%) starting from **19i**; yellow solid; ^{1}H NMR (300 MHz, $CDCl_3$) δ 7.62–7.87 (m, 2H), 7.43–7.52 (m, 2H), 7.00–7.22 (m, 2H) 6.64–6.79 (m, 2H), 4.55–4.73 (m, 2H), 3.84–4.18 (m, 1H), 3.12–3.38 (m, 4H), 2.60–2.77 (m, 7H), 2.38 (*s*, 3H), 1.65–2.22 (m, 10H), 0.92 (t, *J* = 7.2 Hz, 3H). HRMS (*m/z*): [M^+] calcd for $C_{30}H_{40}ClN_4OS$, 539.2611; found, 539.2606.

Compound 18j (GP 1, GP 2, GP 3); yield (29%) starting from **19j**; yellow solid; ^{1}H NMR (300 MHz, $CDCl_3$) δ 7.80–7.88 (m, 2H), 7.43–7.55 (m, 2H), 7.21–7.26 (m, 1H), 6.48–6.65 (m, 3H), 4.53–4.68 (m, 2H), 2.43–3.84 (m, 14H), 1.65–2.19 (m, 10H), 0.92 (t, *J* = 7.2 Hz, 3H); HRMS (*m/z*): [M^+] calcd for $C_{31}H_{42}N_4OSCl$, 553.2768; found, 553.2784.

General Procedure 6 (GP 6):

To a solution of substrate (0.1 mmol) and Et_3N (0.15 mmol) in CH_2Cl_2 (5 mL) was added acyl chloride (0.12 mmol), and the reaction mixture was stirred at room temperature for 3 h. The solvent was removed, and the residue was purified by flash chromatography on silica gel (CH_2Cl_2/MeOH, 30/1) to give the desired product.

Compound 21a (GP 6); yield (86%) starting from **Sant-75** and cyclobutanecarbonyl chloride; yellow solid; ^{1}H NMR (300 MHz, $CDCl_3$) δ 8.67 (br, 2H), 7.27–7.88 (m, 10H), 4.65–4.86 (m, 2H), 3.82–3.98 (m, 1H), 2.90–3.18 (m, 4H), 1.28–2.32 (m, 16H), 0.85 (t, *J* = 7.2 Hz, 3H); HRMS (*m/z*): [M^+] calcd for $C_{35}H_{39}ClN_3O_2S$, 600.2452; found, 600.2437.

Compound 21b (GP 6); yield (91%) starting from **Sant-75** and thiophene-2-carbonyl chloride; yellow solid; ^{1}H NMR (300 MHz, $CDCl_3$) δ 8.70 (br, 2H), 7.01–7.85 (m, 13H), 4.64–4.84 (m, 2H), 3.84–4.04 (m, 1H), 2.88–3.12 (m, 3H), 1.25–2.01 (m, 10H), 0.79 (t, *J* = 7.2 Hz, 3H); HRMS (*m/z*): [M^+] calcd for $C_{35}H_{35}ClN_3O_2S_2$, 628.1859; found, 628.1863.

Compound 21c (GP 6); yield (92%) starting from **Sant-75** and 4-cyanobenzoyl chloride; yellow solid; ^{1}H NMR (300 MHz, $CDCl_3$) δ 8.73 (br, 2H), 8.23 (d, *J* = 7.8 Hz, 2H), 7.39–7.93 (m, 12H), 4.72–4.89 (m, 2H), 3.93–4.10 (m, 1H), 2.95–3.22 (m, 3H), 1.60–1.93 (m, 10H), 0.85 (br, 3H); HRMS (*m/z*): [M^+] calcd for $C_{38}H_{36}ClN_4O_2S$, 647.2247; found, 647.2249.

Compound 21d (GP 6); yield (81%) starting from **Sant-75** and 2-phenoxyacetyl chloride; yellow solid; ^{1}H NMR (300 MHz, $CDCl_3$) δ 8.66 (br, 2H), 6.80–7.90 (m, 15H), 4.64–4.84 (m, 4H), 3.85–3.96 (m, 1H), 2.88–3.07 (m, 3H), 1.45–1.90 (m, 10H), 0.88 (t, *J* = 7.2 Hz, 3H); HRMS (*m/z*): [M^+] calcd for $C_{38}H_{39}ClN_3O_3S$, 652.2401; found, 652.2385.

Compound 21e (GP 6); yield (83%) starting from **Sant-75** and quinoline-2-carbonyl chloride; yellow solid; ^{1}H NMR (300 MHz, $CDCl_3$) δ 8.67 (br, 2H), 7.26–8.25 (m, 16H), 4.67–4.90 (m, 2H), 4.31–4.50 (m, 1H), 3.82–3.91 (m, 1H), 3.24–3.35 (m, 2H), 1.48–2.05 (m, 10H), 0.93 (br, 3H); HRMS (*m/z*): [M^+] calcd for $C_{40}H_{38}ClN_4O_2S$, 673.2404; found, 673.2387.

Compound 21f (GP 6); yield (87%) starting from **Sant-75** and nicotinoyl chloride; yellow solid; ^{1}H NMR (300 MHz, $CDCl_3$) δ 8.67 (br, 4H), 7.19–7.91 (m, 12H), 4.71–

4.88 (m, 2H), 2.95–3.91 (m, 4H), 1.44–2.01(m, 10H), 0.90 (br, 3H); HRMS (*m/z*): [M^+] calcd for $C_{36}H_{36}ClN_4O_2S$, 623.2247; found, 623.2254.

Compound 21g (GP 6); yield (75%) starting from **22** and 2-cyclopentylacetyl chloride; yellow solid; ^1H NMR (300 MHz, $CDCl_3$) δ 8.66 (d, *J* = 4.8 Hz, 2H), 7.35–7.89 (m, 10H), 4.56–5.26 (m, 4H), 3.65–3.72 (m, 2H), 1.06–2.06 (m, 17H); HRMS (*m/z*): calcd for $C_{34}H_{37}ClN_3O_2S$, 586.2295; found, 586.2296.

Compound 21h (GP 6); yield (78%) starting from **22** and 3-phenylpropanoyl chloride; yellow solid; ^1H NMR (300 MHz, $CDCl_3$) δ 8.66 (d, *J* = 4.8 Hz, 2H), 7.14–7.86 (m, 15H), 4.67–4.82 (m, 2H), 3.65–3.72 (m, 2H), 2.85–2.94 (m, 2H), 2.36–2.45 (m, 2H), 1.73–1.96 (m, 8H); HRMS (*m/z*): [M^+] calcd for $C_{36}H_{35}ClN_3O_2S$, 608.2139, found, 608.2134.

Compound 21i (GP 6); yield (80%) starting from **22** and 2-(3-chlorophenyl)acetyl chloride; yellow solid; ^1H NMR (300 MHz, $CDCl_3$) δ 8.66 (d, *J* = 4.8 Hz, 2H), 7.10–7.86 (m, 14H), 4.82–5.80 (m, 4H), 3.64–3.73 (m, 2H), 1.67–1.90 (m, 8H); HRMS (*m/z*) calc. for $C_{35}H_{32}Cl_2N_3O_2S$, 628.1592; found, 628.1594.

Compound 21j (GP 6); yield (87%) starting from **22** and 2-phenoxyacetyl chloride; yellow solid; ^1H NMR (300 MHz, $CDCl_3$) δ 8.66 (d, *J* = 4.8 Hz, 2H), 6.28–7.86 (m, 15H), 4.70–4.85 (m, 2H), 4.40 (s, 2H), 3.71–3.85 (m, 2H), 1.65–1.95 (m, 8H); HRMS (*m/z*): [M^+] calcd for $C_{35}H_{33}ClN_3O_3S$, 610.1931; found, 610.1943.
